# Ingenol mebutate–associated immune thrombocytopenic purpura

**DOI:** 10.1016/j.jdcr.2024.01.028

**Published:** 2024-02-07

**Authors:** Hafsa Zuberi, Sanober A. Amin

**Affiliations:** a Texas Tech University Health Sciences Center School of Medicine, Lubbock, Texas; b Dermatology Solutions, Grapevine, Texas

**Keywords:** actinic keratosis, drug recall, immune thrombocytopenic purpura, ingenol mebutate

## Introduction

Ingenol mebutate (Picato gel, LEO Pharma, Inc) is a topical diterpene ester that was approved for the treatment of actinic keratoses (AK) by the US Food and Drug Administration in 2012. In addition, it has been used off-label for anogenital warts.^1^ It is derived from the sap of the *Euphorbia peplus* plant and has historically had therapeutic use for a variety of skin conditions. The drug causes rapid necrosis in as little as 1 day after application by increasing intracellular and mitochondrial calcium, triggering cellular swelling and loss of membrane integrity. Furthermore, ingenol mebutate can cause a delayed neutrophil-mediated cellular oxidative burst and antibody-mediated cytotoxicity response via specific activation of immune pathways involving protein kinase C delta.^2^, ^3^, ^4^ Commonly reported side effects of ingenol mebutate use include localized pruritus, erythema, crusting, scaling, and ulceration.^3^^,^^5^

Following reports of severe adverse reactions, the Food and Drug Administration issued a warning in 2015 detailing the risk of severe hypersensitivity reactions, herpes zoster, allergic contact dermatitis, and eye injuries following ingenol mebutate use. The medication was eventually withdrawn by LEO Pharma in 2020 from worldwide markets due to an increased risk of squamous cell carcinoma and other nonmelanoma skin malignancies associated with the drug’s use when compared to other treatment options of AKs.^5^, ^6^, ^7^ While the marketing authorization for ingenol mebutate was withdrawn in the European Union in February 2020, as of the date of publication of this report, no similar withdrawal has been announced by the Food and Drug Administration.

We describe an unreported, potentially life-threatening systemic adverse reaction from the use of topical ingenol mebutate. This is of clinical relevance as patients may still be in possession of it despite the recall, and can potentially experience severe an adverse reaction when the drug is used without the knowledge of the prescribing physician.

## Case report

**Fig 1:**
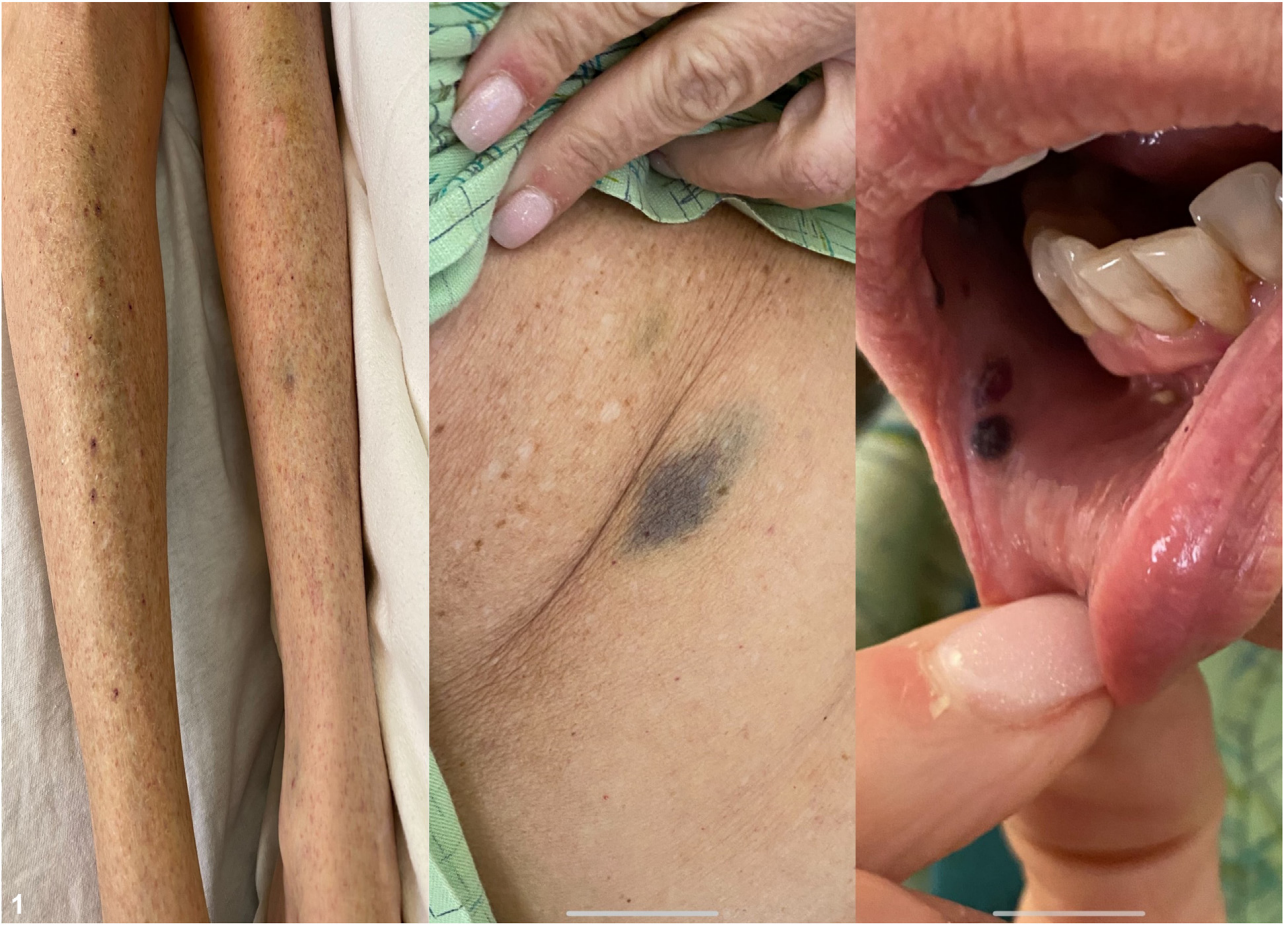
Manifestations of immune thrombocytopenic purpura after application of ingenol mebutate. Purpura and petechiae present on the patient’s legs and underarms and inside of her mouth.

A 62-year-old Caucasian female presented in early April 2021 with widespread petechiae, purpura, and ecchymoses on her trunk, extremities, as well as mucosal purpura on buccal mucosa, 6 days after applying ingenol mebutate gel on her chest AKs for the prescribed duration of 3 days [Fig 1]. She was prescribed the gel in September 2020 by her former dermatology practice and started use in late March 2021, unaware of the recall. She did not experience any cutaneous adverse reactions on the treatment area; however, she reported immediate onset of headache on treatment day and oral mucosal purpura on the fifth day, which progressed to ecchymoses on her trunk and extremities on the sixth day. Her past medical and surgical history included anxiety, hypercholesterolemia, hypothyroidism, appendectomy, and lumpectomy of breast. Her dermatologic history included 3 squamous cell carcinomas in situ on her left leg treated with topical or intralesional 5-fluorouracil, with localized inflammation only. Medications and supplements included Armour Thyroid, vitamin D, zinc sulfate, and azelastine. She denied any history of autoimmune disease, hepatic disease, splenic enlargement, cardiac disease, alcoholism, recent infections, hematuria, hematochezia, or gingival bleeding. Complete blood count (CBC) on the day of presentation showed a platelet count of 5000/uL, compared to her baseline complete blood count in August 2020 with a platelet count of 348,000/uL. Urinalysis was positive for occult blood.

She was admitted to the hospital for severe thrombocytopenia under the working diagnosis of immune thrombocytopenic purpura attributed to ingenol mebutate use. She was treated with systemic steroids and platelet transfusions which improved her platelet count to 37,000/uL after 4 days. Extensive postdischarge hematology/oncology and rheumatology workup including a bone marrow biopsy was negative for any hematological pathology, autoimmune condition, or malignancy. Since the time of initial presentation and treatment, the patient has undergone follow-up with dermatology and rheumatology; her platelet counts have returned to baseline and there has been no further incidence of thrombocytopenia.

## Discussion

Commonly reported side effects of ingenol mebutate include localized erythema, crusting, scaling, ulceration and pruritus, and systemic hypersensitivity reactions.^3^, ^4^, ^5^ There have been reports of new onset pemphigus erythematosus and herpes zoster and other latent viruses, as well as relapsed pemphigus vulgaris with ingenol mebutate use due to it mediating a blistering response.^8^^,^^9^ In addition, its use has been linked to increased rates of developing squamous cell carcinoma and rapidly growing keratoacanthomas, when compared to other approved treatments of AK. European Medicines Agency cited that 3.3% of patients receiving ingenol mebutate developed a skin malignancy vs 0.4% of patients treated with the comparative agent imiquimod.^6^ Although it displayed a high clinical efficacy in treatment of AK, ingenol mebutate is believed to activate prooncogenic molecular signaling pathways that cause cytological atypia and hence malignancy.^5^

We present a unique case of a systemic adverse drug effect attributed to topical ingenol mebutate application, highlighting the clinical relevance of topical medications causing immune activation and yielding systemic reactions despite a theoretically low transcutaneous absorption. Our patient presented with significant thrombocytopenia after localized use of a drug, implying that even minimal cutaneous absorption could cause significant systemic effects. Many drugs have been implicated in the development of immune thrombocytopenic purpura, with the most common being heparin (heparin induced thrombocytopenia); however, there have been no reports in literature thus far of ingenol mebutate causing immune thrombocytopenic purpura. Clinicians should be aware of this rare but serious potential side effect of this medication, as patients may still be in possession of it despite the recall.

## Conflicts of interest

None disclosed.
